# Assessing Text Experience in British Primary School Children: New Validated Title and Author Recognition Tests

**DOI:** 10.1177/17470218261421104

**Published:** 2026-01-23

**Authors:** Holly Cooper, Maria Korochkina, Marc Brysbaert, Kathleen Rastle

**Affiliations:** 1 Department of Psychology, Royal Holloway, University of London, Egham, UK; 2 Department of Experimental Psychology, Ghent University, Belgium

**Keywords:** text experience, author recognition test, title recognition test, reading, spelling

## Abstract

Becoming a skilled reader requires that children accumulate extensive experience with text through independent reading. Research shows that greater text experience is associated with stronger reading skills, better comprehension, and improved spelling, and, consequently, higher reading motivation. Reliable objective measures of children’s reading experience are therefore essential; however, because such measures are typically highly sensitive to temporal and cultural contexts, none of the existing tests is suitable for capturing the reading experience of British children today. We address this gap by introducing a new Author Recognition Test and Title Recognition Test designed specifically for primary school children in the United Kingdom and validated with a large cohort of British pupils. The battery also includes a new multiple-choice spelling test that can be easily administered online. We further demonstrate that single-word reading and sentence reading efficiency tests from the Rapid Online Assessment of Reading can be adapted for use with British children and provide valid measures of reading proficiency. Together, these tools offer a much-needed, freely available resource for both researchers and practitioners, enabling reliable measurement of children’s text experience and basic literacy skills. The test battery is openly available on https://osf.io/gmv72/.

## Introduction

Reading is the foundation for success in education, the workplace, and active participation in society, and learning to read is the key objective of primary education. Extensive research has examined how children acquire reading skills and the most effective ways to teach them (see, e.g., Castles et al., 2018, for a comprehensive review). This research unequivocally demonstrates that learning to read in an alphabetic writing system begins with understanding the relationship between written symbols and the sounds of spoken language, and that explicit instruction underpins this learning (Rastle et al., 2021). However, becoming a skilled reader involves more than decoding print into sounds; it requires developing the ability to access word meanings directly from print (Castles et al., 2018). Research shows that the shift to this advanced form of word recognition depends on children’s independent reading (e.g., Share, 2004), as only extensive experience with text allows emerging readers to encounter words repeatedly and in varied contexts (e.g., Castles et al., 2007; Korochkina et al., 2024; Mol & Bus, 2011; Nation, 2017). Yet, measuring a child’s reading experience remains challenging. In this article, we introduce a new measure of text experience validated with a sample of British primary school children.

Research suggests that substantial individual variation in text experience begins before the point at which children can read independently, and that it is associated with reading outcomes. Early text experience during emergent literacy typically takes place in shared reading of picture books, when children hear text read aloud while also seeing the written words. Picture books have been shown to provide much richer language than everyday child-directed speech (e.g., Dawson et al., 2021; Green et al., 2023), and modelling studies suggest that children who regularly engage in shared reading are exposed to millions more words than those whose parents read to them less often (e.g., Green et al., 2023; Hart & Risley, 2003; Logan et al., 2019; Montag et al., 2018). Research further shows that while 3- to 5-year-olds primarily focus on illustrations, children’s attention to print increases with age—especially when adults point to the text (Evans et al., 2008). Consistent with these findings, meta-analyses demonstrate that shared book reading accounts for about 8% of the variation in language and reading performance among children under 8 years old (Bus et al., 1995), and that the amount of shared reading predicts about 12% of the differences in preschoolers’ oral language skills (Mol & Bus, 2011).

This relationship between text experience and literacy outcomes also applies to children’s independent reading. Meta-analyses show moderate to strong correlations between text experience and reading skills from early childhood through young adulthood (2–21 years) and indicate that frequent reading has a lasting, positive impact on academic success (Mol & Bus, 2011). These findings support a developmental model of reading in which text experience plays a crucial role in shaping literacy (Mol & Bus, 2011). Critically, this relationship has been shown to be reciprocal: increased text experience improves reading skills, making reading more enjoyable, which in turn motivates children to read more and further enhance their reading ability (Leppänen et al., 2005; Mol & Bus, 2011; van Bergen et al., 2018). In contrast, readers with less text experience have fewer opportunities to develop their reading skills, are more likely to find reading challenging, and consequently are less motivated to read, thereby widening the gap between weaker and stronger readers (Allington, 1984; Mol & Bus, 2011). This phenomenon is known as the “Matthew effect” in reading (Bast & Reitsma, 1998; Foster & Miller, 2007; Mol & Bus, 2011; Stanovich, 1986).

The importance of text experience in reading acquisition has motivated research to develop the means to measure children’s text experience. One type of measure relies on subjective judgements, such as questionnaires, interviews, or self-report diaries, in which children or parents estimate or record reading activity. However, these measures are often limited by social desirability bias: because reading is generally viewed positively, responses tend to be over-reported (e.g., Brysbaert et al., 2020; Chateau & Jared, 2000; Masterson & Hayes, 2007). These measures also often rely on time-based responses such as “less than 30 minutes” or “2 hours or more” (e.g., Mullis et al., 2012), which can be difficult for children to understand and therefore threaten the validity of the instrument.

Consequently, increasing efforts have focused on developing *objective* methods for measuring text experience. The most commonly used of these are the Author Recognition Test (ART; Stanovich & West, 1989) and the Title Recognition Test (TRT; Cunningham & Stanovich, 1990). These tests present participants with a list containing real authors’ names (ART) or book titles (TRT) alongside made-up names (foils), and participants indicate which authors or titles they recognise by ticking the corresponding boxes. These tests are based on the assumption that frequent readers will have encountered more books and authors and thus will recognise more items (Huang & Bolt, 2024). The key advantage of tests such as the ART and the TRT is that they minimise social desirability bias while remaining quick to administer and easy to understand (Brysbaert et al., 2020; Cunningham & Stanovich, 1990; Mol & Bus, 2011; Stainthorp, 1997; Stanovich & West, 1989). However, a key challenge with the TRT and ART is their sensitivity to time and place: authors and books familiar to readers at one point in time may be unfamiliar just a few years later, and familiarity can also vary widely across countries, even within the same language (Stainthorp, 1997; Su et al., 2024; Wimmer & Ferguson, 2023). Several tests have been developed, but none adequately captures the reading experience of British children today: they are either designed for adults (Wimmer & Ferguson, 2023), are unsuitable for the United Kingdom (Brysbaert et al., 2020; Chen & Fang, 2016; Zhang et al., 2018), or are outdated (Masterson & Hayes, 2007; Ricketts et al., 2007; Stainthorp, 1997). The aim of our study was therefore to address this gap by introducing new ART and TRT tests designed specifically for British primary school children.

The development of a psychometric test requires careful validation; however, the application of validation procedures in cognitive psychology remains inconsistent (see Brysbaert, 2024, for an overview of key principles for developing and evaluating psychometric tests and commentary on their variable application within the field). In our work, we followed established best practices to ensure that our tests accurately capture the full spectrum of performance, consistently assess a single skill, and reliably measure differences between participants over time. A critical part of this process is confirming that the test genuinely evaluates the intended skill or ability, thereby establishing its construct validity. Typically, this involves examining correlations with established assessments of related traits. Research indicates that the key literacy skills expected to improve with increased text experience are word reading, reading comprehension, and spelling. For example, Cipielewski and Stanovich (1992) demonstrated that third- to fifth-grade students’ (ages 8–11) performance on the ART and TRT was strongly correlated with their reading rate and comprehension. McBride-Chang et al. (1993) found similar results for fifth- to ninth-grade participants (ages 10–15), showing positive relationships between TRT performance and word reading, comprehension, and vocabulary, especially among lower ability readers. Spear-Swerling et al. (2010) reported that ART performance was positively associated with word reading, comprehension, and vocabulary in sixth graders (ages 11–12). Likewise, Ecalle and Magnan (2008) observed positive relationships between TRT and ART performance and spelling abilities in first graders, with cross-sectional data showing the same pattern in fourth and fifth graders. Cunningham and Stanovich (1990) similarly reported strong correlations between TRT scores and spelling abilities in children from fourth to sixth grade (ages 9–12).

Based on this literature, we validated our new test battery using two assessments of reading skill—single-word reading and sentence reading efficiency—along with a spelling assessment. We also examined the relationship between test performance and age, as research indicates that text experience generally increases with age, with older children having more cumulative reading experience (e.g., Choi et al., 2017; Grolig et al., 2020; Liu et al., 2016; Payne et al., 2014).

## Method

Test development was conducted in two stages. Stage 1 included creating a large pool of items and administering them to a cohort of pupils. Based on their performance, we applied selection criteria to decide which items to retain and which to exclude. This decision was informed by how well pupils recognised the items and how consistently each item aligned with the rest of the test. Our goal was to develop a test that included items of varying difficulty and was capable of discriminating between pupils with different levels of text experience. After retaining the best-performing items and removing the less effective ones, stage 2 included administering the revised tests to a different cohort of pupils. Using data from this new sample, we assessed the reliability of the tests. Finally, we validated the tests by examining whether pupil TRT and ART performance was associated with reading and spelling ability, and age.

### Stage 1

#### Participants

One hundred and twenty-three pupils in England’s key stage 2 (school years 3–6; ages 7–12 years; mean age = 9 years, *SD* = 1.18 years; 68 females, 52 males, 3 preferred not to disclose their gender) took part in stage 1. Pupils were recruited from a primary school in Hertfordshire. Parents provided opt-out consent, and children gave verbal assent before the start of the testing. The school was compensated for its assistance with the study through a small payment in Amazon vouchers. The study received ethical approval from the Research Ethics Committee at Royal Holloway, University of London (Ref. 305).

#### Materials

One hundred book titles and 100 authors were selected from two main sources: (a) the Children and Young People’s Books Lexicon (CYP-LEX; Korochkina et al., 2024) and (b) the ‘What Kids are Reading’ reports from Renaissance (Topping et al., 2021, 2022, 2023). To ensure the TRT was measuring *text* experience, any titles that were adapted for TV or film post-1999 were excluded (e.g., the *Harry Potter* series). In addition, because many books are published as part of a series (e.g., *Skulduggery Pleasant*, which includes 18 separate titles), we used the series name (e.g., *Skulduggery Pleasant*) or the most popular title from a series (e.g., *The 13-Storey Treehouse* title from *The Treehouse* series). For each of the ART and the TRT, 30 foils were added. Twenty-seven of the ART foils were taken from Ricketts et al. (2007), and the remaining three were created by randomly pairing first and last names. Following Zhang et al. (2018), foils for the TRT were created by changing one word in a real book title (e.g., *The Plague Dogs* became *The Gloomy Dogs*).

#### Procedure

Participants completed the TRT and ART tests via Qualtrics software (Qualtrics LLC), with items presented in a random order for each participant. They were told that some authors’ names and book titles were real and some were made-up and instructed to tick boxes next to those authors and titles that they recognised, and to be careful not to tick boxes next to made-up authors’ names and titles. On average, 17% of book titles and 13% of authors’ names were recognised, with minimal foil selection (1%–2%).

#### Analysis

As we argued in the Introduction, a key aspect of test development is ensuring the items are appropriate and measure the intended construct (here, text experience). Thus, in line with Brysbaert’s (2024) recommendations, we conducted item validation using two key criteria—mean recognition rate and item-rest correlation (IRC)—to determine which items to retain for the final tests. In addition, we applied item response theory (IRT; Embretson & Reise, 2000) to model how participants with varying abilities would respond to individual test items. Once the final set of test items was validated, we assessed the overall reliability of the tests using measures of internal consistency to ensure all items measured the same construct. The following paragraphs detail the procedures used to conduct item validation and assess test reliability.

Mean recognition rate indexes the percentage of participants who recognised an item. Items with higher recognition rates are considered “easy” (e.g., *Roald Dahl* was recognised by 77% of participants), whereas those with lower recognition rates are considered “difficult” (e.g., *Roderick Hunt* was recognised by 1% of participants). IRCs indicate how well an item fits with the overall test by measuring the correlation between this item and the rest of the items (e.g., Henrysson, 1963). Higher positive IRCs indicate stronger consistency with the rest of the test. In contrast, items with low IRC values are considered to have poor consistency with the other items, suggesting that they may measure a different construct and should be removed. We combined the two criteria—mean recognition rate and IRCs—in the following way: “easy” items (i.e., those recognised by 23%–77% of participants) were retained if their IRC score was above 0.2, while “difficult” items (i.e., those recognised by 5%–22%) were retained if their IRC score was above 0.3. This approach ensured that the test included only those items that were consistent with the rest, while still varying in difficulty. In addition, one title (*A Good Girl’s Guide to Murder*) was removed as it was adapted into a TV show shortly after testing and, as a result, we could not be certain that recognition was due to text experience alone. The final version of the TRT included 80 items (50 real items and 30 foils), and the final version of the ART included 77 items (47 real items and 30 foils).

Item retention based on mean recognition rate and IRC was followed by IRT analysis to evaluate how well the selected test items discriminated between different levels of ability. In applying IRT to determine which items to retain, we followed the methodology established in earlier research (e.g., McCarron & Kuperman, 2021; Moore & Gordon, 2015). The core premise of this approach is that the probability of a correct response is shaped by two key item properties: item difficulty and item discrimination. An ideal test should include variation in difficulty and good discrimination. The *difficulty* parameter reflects the level of ability needed for a 50% chance of responding correctly, and more difficult items are associated with more positive values. The typical range for this parameter is between −2 (very easy) and 2 (very difficult) (Hays et al., 2000; Ranyard et al., 2020). The *discrimination* parameter reflects how effectively an item distinguishes between individuals with low and high ability levels; higher values suggest that an item is better at differentiating between abilities. This parameter ideally is larger than 0.5 (Hays et al., 2000; Ranyard et al., 2020). Across the TRT and ART, item difficulty ranged between −1.48 and 2.52, and item discrimination ranged between 0.37 and 4.60, indicating that the items in both tests varied in difficulty and effectively discriminated among different ability levels in this sample.

Finally, to assess the reliability of the two tests, we evaluated their internal consistency, which reflects the extent to which the items within a test measure the same underlying construct (see Brysbaert, 2024, for a recent discussion of approaches to assessing internal consistency). We calculated two coefficients: Cronbach’s alpha (α; Cronbach, 1951) and McDonald’s omega (ω; McDonald, 1999). *Cronbach’s alpha* is the most commonly used internal consistency coefficient and is based on the average inter-item correlations (Dunn et al., 2014). However, it is sensitive to the number of items in a test and can underestimate or overestimate reliability when item loadings vary. *McDonald’s omega* addresses these limitations by relying on factor analysis, in which the sum of all factor loadings and error variances is used (Orçan, 2023). Generally, internal consistency is considered acceptable when both α and ω exceed .70 (DeVellis & Thorpe, 2003; Dunn et al., 2014; Tavakol & Dennick, 2011). In our study, both coefficients were .90 or above for the TRT (α = .90, ω = .91) and the ART (α = .91, ω = .92), indicating that both tests demonstrated high reliability in this sample of pupils.

### Stage 2

Once we had revised the tests and ensured they were appropriate based on data from the first cohort of pupils, we administered them to a new sample, alongside a series of tasks measuring reading and spelling ability. We assessed the reliability of the revised tests using the same internal consistency coefficients as before, now applied to the data from the new sample. Next, we analysed the association between pupils’ TRT and ART scores and their reading and spelling performance and then examined the relationship between TRT and ART performance and age. In this section, we first describe the new sample, then detail the construction, administration, and scoring of each test, outline our analytical approach, and finally report the results of all analyses.

#### Participants

Participants were 130 pupils in England’s key stage 2 (school years 3–6; ages between 7 and 11 years) recruited from 2 primary schools in Hertfordshire (mean age = 9 years; *SD* = 1.14 years; 73 female, 49 male, 8 preferred not to disclose their gender). In this cohort, 12 participants were 7 years old, 32 were 8 years old, 41 were 9 years old, 31 were 10 years old, and 14 were 11 years old. Two additional participants started the tests but were unable to complete them due to poor reading proficiency. There were also task-specific exclusions: one participant did not complete the spelling task, and three participants did not understand the sentence reading task. Opt-out consent was obtained from parents, and children were provided multiple opportunities to provide positive assent. The schools received a small payment in the form of Amazon vouchers for their assistance with the study. The study received ethical approval from the Research Ethics Committee at Royal Holloway, University of London (Ref. 305).

#### Materials

All materials and a link to the fully programmed experiment are available at https://osf.io/gmv72/.

##### Final Versions of the TRT and ART

The scoring of the TRT and ART followed previous research (Brysbaert et al., 2020; McBride-Chang et al., 1993; Stainthorp, 1997): scores were calculated as the percentage of real book titles and authors’ names selected minus the percentage of foils selected. For example, if a participant selected 20 of the 50 real titles and 2 of the 30 foils, their corrected score was 33.3%, calculated as: ((20/50) × 100) − ((2/30) × 100). The final versions of the tests, along with the exact test instructions, are provided in Supplemental Table S1 (also available in this project’s repository on OSF, under *All instructions and stimuli*; see https://osf.io/gmv72/).

##### Single Word Reading

We used the Lexical Decision Task from the Rapid Online Assessment of Reading Ability (ROAR-LDT) as a measure of single-word reading proficiency (Yeatman et al., 2021). In this assessment, participants are presented with letter strings (including real words and nonwords) and must decide whether each string is a real English word. The test comprises three lists, with each participant randomly assigned to complete one list (84 items per list: 42 real words, 42 nonwords). Full stimuli lists are available in Yeatman et al. (2021). The task begins with 10 practice trials (5 words, 5 nonwords), with feedback provided for each.

We adapted six aspects of the original ROAR-LDT assessment to ensure its suitability for British children and our specific testing setting: (a) nonwords were described as “made-up” instead of “magical”, (b) the game element (collecting coins) was removed, (c) each stimulus remained on the screen until a response was given, rather than disappearing after 350 ms as in the original version of the test, to accommodate younger participants with lower reading abilities, (d) each trial automatically advanced to the next after 4 s, rather than having no time limit as in the original version, to account for the increased stimulus presentation time, (e) participants used the “A” and “L” keys to respond, instead of the left and right arrow keys, and (f) two breaks were included after 28 consecutive trials. To avoid confusion about which key to press, the A button was covered with a red circle sticker, and the L button with a green circle sticker. Participants were instructed to press the “red button” if they believed a word was made-up and the “green button” if they believed a word was real. The task took approximately 5 min to complete, and scores were calculated based on the total number of correct responses out of 84 (Yeatman et al., 2021).

##### Sentence Reading Efficiency

We used the ROAR-Sentence task (Yeatman et al., 2024) as a measure of sentence reading efficiency. This task requires participants to read as many sentences as possible and label them as true or false in 3 min. The task included 130 sentences. An example of a true sentence is “You can read stories at school”, and an example of a false sentence is “An apple is blue”. The task started with four practice trials (two true, two false), and feedback was provided for each. We made two adaptations from the original: (a) language was adapted to be more appropriate for British children (e.g., “trash” and “soccer” were changed to “rubbish” and “football”), and (b) participants used “A” and “L” instead of the left and right arrow keys. We refer the reader to Yeatman et al. (2024) for the original stimuli list (https://github.com/yeatmanlab/ROAR-SRE-Public) and to Supplemental Table S2 (https://osf.io/gmv72/) for our adapted sentences. Like in the ROAR-LDT task, the A button was covered with a red circle sticker, and the L button with a green circle sticker. Participants were instructed to press the “red button” if they believed a sentence was false and the “green button” if they believed a sentence was true. The task took approximately 5 min to complete. The task assessed how many trials participants could complete in 3 min, and, to control for guessing, scores were calculated as the total number of correct responses minus the total number of incorrect responses (Yeatman et al., 2024).

##### Spelling Ability

Existing spelling tests typically require children to write or type responses. To reduce the time taken for the test, we created a multiple-choice spelling test that could be administered online. Twenty items were selected from a larger list of spelling words from Spelling Frame (an online resource with various spelling games and lists structured according to England’s spelling curriculum; https://spellingframe.co.uk/). Each item included the word itself (e.g., *soldier*) and a carrier sentence (e.g., “Tim’s great grandfather was a *soldier* in the second world war”) to provide context for its usage. A recording of both the word and the carrier sentence was made by the first author. Each recording had the following structure: the word in isolation, followed by the word in a carrier sentence, and then the word in isolation again, with 1 s of silence at the start, between each part, and at the end. The resulting recordings were 8 to 10 s long. Participants were asked to listen to the recordings and select the correct spelling from four options: one correct spelling, and three incorrect options, each presenting an alternative way to spell the given sound sequence. For example, the four options for the word *soldier* were *soldier*, *soldjer*, *soldger*, and *solger* (the full list of stimuli is provided in Supplemental Table S3; see https://osf.io/gmv72/). There were four practice trials with feedback. The task took between 5 and 10 min to complete, and scores were calculated based on the total number of correct responses out of 20.

#### Procedure

The single-word reading, sentence reading efficiency, and spelling tasks were created and hosted through Gorilla (https://www.gorilla.sc/), and the participants were redirected to Qualtrics software (Qualtrics LLC) for the ART and TRT. Participants were tested in groups of four, and the order of the tasks was consistent across all participants: the TRT and ART, followed by the reading tasks (ROAR-LDT and ROAR-Sentence), and finally the spelling task. The ART and TRT were verbally explained to participants to ensure full understanding. The instructions for the reading and spelling tasks were displayed on the screen and narrated through headphones to help any struggling readers. The whole experiment took less than 30 min to complete.

#### Analysis

The data were analysed in R, version 4.4.1 (R Core Team, 2024). For the ROAR-LDT task, prior to conducting the inferential analysis, we inspected the response times (RTs) and removed all data points which fell outside the distribution (i.e., responses faster than 200 ms and slower than 3,000 ms, which resulted in loss of less than 1% of data). Only RTs for correct responses were used. In line with Yeatman et al. (2021) and supported by the Box–Cox test (Box & Cox, 1964), we used median log-transformed RTs in all analyses.

Three analyses were conducted to explore the reliability and validity of the TRT and ART: first, we assessed the tests’ internal consistency using Cronbach’s α and McDonald’s ω; second, we computed correlations between the pupils’ performance on the TRT and ART and their performance on the reading and spelling tasks (all accuracy scores were standardised using z-score transformation); and, finally, simple linear regressions were used to examine the relationship between task performance (raw scores) and age.

## Results

**Figure 1.**
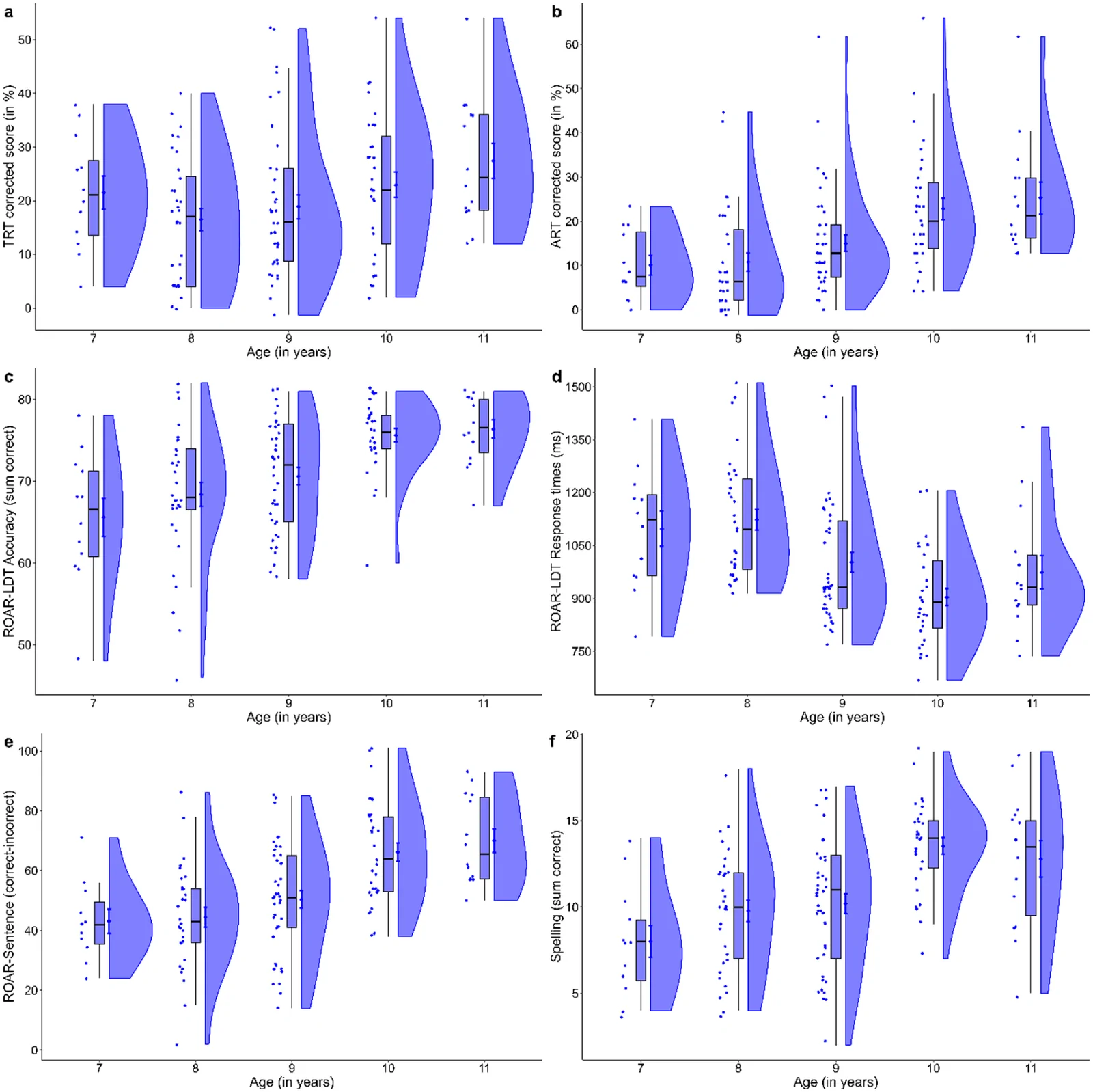
Participants’ performance on the TRT (a), ART (b), ROAR-LDT accuracy (c), ROAR-LDT response times (d), ROAR-Sentence (e), and spelling (f) as a function of age. The dots represent individual data points, the boxes and whiskers indicate the four quartiles of the ordered data, the black horizontal lines mark the medians, and the shaded clouds show the estimated data distributions, together with the means (the dots positioned on the clouds) and one standard error of the mean (the blue error bars around the blue dots). Note that age is used as a continuous predictor in the analyses but plotted here as a factor (7–11 years old) to provide a clearer visual interpretation. *Note.* ART = author recognition test; TRT = title recognition test; ROAR-LDT = lexical decision task from the rapid online assessment of reading ability.

The data from each test across the different ages are presented in Figure 1. Although there is considerable variability in performance within each age group, the figure shows that, overall, pupils’ performance improved with age: average scores on all tasks increased, while response times on the ROAR-LDT decreased. In the remainder of this section, we first report the results of our analyses on the internal consistency of TRT and ART, as well as the relationships between all tasks, and then present the statistical analysis examining the relationship between task performance and age.

### Test Reliability

Cronbach’s α and McDonald’s ω for each test are reported in Table 1. All tasks showed acceptable to excellent internal consistency, indicating that they reliably measured the same underlying construct.

**Table 1.** Internal Consistency Coefficients (Cronbach’s α and McDonald’s ω) for Each Task. Values Above .70 Are Considered Acceptable.

Task	Cronbach’s α	McDonald’s ω
TRT	.87	.88
ART	.89	.91
ROAR-LDT		
List 1	.83	.86
List 2	.88	.90
List 3	.86	.89
ROAR-Sentence	.96	.96
Spelling	.74	.77

*Note.* ART = author recognition test; TRT = title recognition test; ROAR-LDT = lexical decision task from the rapid online assessment of reading ability.

### Relationship Between Tasks

Correlational analyses on *z*-transformed scores were conducted between all tasks, and the resulting correlation coefficients are reported in Table 2. There was a strong positive relationship between ART and TRT performance, suggesting that they largely measure the same construct. Both tasks also showed significant positive associations with performance on single-word reading accuracy (ROAR-LDT accuracy), sentence reading efficiency (ROAR-Sentence), and spelling (all *p*’s <.001). In addition, superior performance on the ART was associated with faster responses on the single-word reading task (ROAR-LDT RT; *p* < .001), but no such relationship was observed for the TRT (*p* = .054).

**Table 2.** Correlation Matrix Displaying the Relationship Between TRT, ART, ROAR-LDT Accuracy, ROAR-LDT RT, ROAR-Sentence, and Spelling. All Correlations Were Significant (*p* < .001), Except for That Between TRT and ROAR-LDT RT (*p* = .054, *N* = 130).

Task	TRT	ART	ROAR-LDT		ROAR-Sentence
			Accuracy	RTs	
ART	.79				
ROAR-LDT					
Accuracy	.30	.45			
RTs	−.17	−.29	−.35		
ROAR-Sentence	.47	.58	.72	−.45	
Spelling	.32	.49	.64	−.39	.62

*Note.* ART = author recognition test; TRT = title recognition test; ROAR-LDT = lexical decision task from the rapid online assessment of reading ability; RT = response time.

### Relationship with Age

We fit six simple linear regression models—one for each test—using age as the (continuous) independent variable and task performance as the dependent variable. The output of all models is reported in Table 3. Age had a significant effect in all models, suggesting that older children performed better on each task.

**Table 3.** Output of the Linear Regression Analyses Examining the Relationship Between Age and Task Performance for Each Task. The Letter β Denotes Standardised Coefficients.

Task	β	SE	*t*	*p*
TRT	2.25	1.00	2.25	.026
ART	4.67	0.94	4.95	<.001
ROAR-LDT				
Accuracy	3.02	0.51	5.90	<.001
RTs	−.07	0.01	−5.03	<.001
ROAR-Sentence	8.41	1.36	6.16	<.001
Spelling	1.43	0.27	5.31	<.001

*Note.* ART = author recognition test; TRT = title recognition test; ROAR-LDT = lexical decision task from the rapid online assessment of reading ability; SE = standard error.

## Discussion

Research demonstrates that reading experience is crucial for the transition from novice to skilled reader, as it repeatedly exposes children to a wide range of words across diverse contexts (e.g., Castles et al., 2007; Korochkina et al., 2024; Mol & Bus, 2011; Nation, 2017). Stronger reading skills make reading more enjoyable and motivate children to engage more frequently with texts, creating a positive feedback loop that further enhances reading ability (e.g., Leppänen et al., 2005; van Bergen et al., 2018). Consequently, there has been considerable interest among researchers in developing objective and reliable measures of reading experience. This body of research indicates that TRT and ART are up to the challenge; however, their sensitivity to temporal and cultural context means that they require regular updating to remain appropriate for different populations and regions, even within the same language (e.g., Stainthorp, 1997; Su et al., 2024; Wimmer & Ferguson, 2023). As a consequence, no existing test can adequately capture the reading experience of contemporary British children. The research reported here addresses this gap by developing a new validated test battery specifically designed for primary school children in the United Kingdom.

Our test battery comprises TRT and ART, developed using a rigorous procedure to select items that are age-appropriate, able to discriminate readers at different points along the text experience spectrum, and internally consistent, ensuring that each test measures a single underlying construct (see Brysbaert, 2024, for an overview of best practices in test validation). To ensure that our tests accurately capture pupils’ text experience, we drew on years of research demonstrating that text experience is positively correlated with reading and spelling skills, as well as age (e.g., Cipielewski & Stanovich, 1992; Ecalle & Magnan, 2008; McBride-Chang et al., 1993; Spear-Swerling et al., 2010). In our study, this involved examining the correlations between children’s performance on the TRT and the ART and their accuracy and response times on the lexical decision task (ROAR-Word; Yeatman et al., 2021), as well as the correlations between TRT and ART scores and children’s sentence reading efficiency (ROAR-Sentence; Yeatman et al., 2024), spelling ability, and age.

These analyses yielded results in the expected direction and aligned with previous research, supporting the validity of our tests as measures of text experience. Pupils who recognised more titles and authors made fewer errors when recognising words (with those recognising more authors also responding more quickly) were more efficient at reading sentences and judging their meaningfulness and demonstrated stronger spelling skills. Performance on all tasks also improved with age, suggesting that our tests were sufficiently sensitive to detect differences in text experience both within and across age groups. Importantly, children’s performance on the TRT and ART was strongly positively correlated, with the correlation between the two tests approaching the internal consistency of each test (i.e., correlations across test items). Together, these results indicate that both tests are suitable tools for estimating the amount of text experience among British primary school children.

In addition to providing evidence that our tests are valid measures of text experience, our findings offer important insights into children’s reading. One notable finding is that, compared to the TRT, the ART showed a stronger association with reading and spelling ability as well as age. Previous research has generally assumed the opposite, as children are thought to be more likely to remember a book title than an author’s name (Hug et al., 2024), and a study of American fourth- to sixth-grade students (ages 9–12) supported this assumption (Echols et al., 1996). We believe the difference between our findings and previous results lies in the nature of our tests, which were specifically designed to reflect current reading practices in the United Kingdom. In our tests, titles of the most popular books (e.g., the *Harry Potter*, *Percy Jackson*, and *Diary of a Wimpy Kid* series) were excluded, while the names of the corresponding authors (J.K. Rowling, Rick Riordan, and Jeff Kinney) were included. This decision was made because many of these titles have been adapted for film or TV, meaning that familiarity with them may not necessarily reflect *reading* experience. Furthermore, recent analyses of reading habits in the United Kingdom suggest that children tend to read books within a series (e.g., all *Diary of a Wimpy Kid* books) rather than across different series or authors (e.g., Topping et al., 2021, 2022, 2023). Therefore, while our results do not suggest that children are more familiar with authors than titles, they indicate that, due to the current patterns in reading habits, the ART may be a more sensitive measure of text experience than the TRT for contemporary British primary school children. One practical implication of this finding is that, compared with the TRT, the ART may have greater longevity and require less frequent updating, as authors typically remain relevant for longer, particularly if they continue publishing popular books or series.

Another notable contribution of this work is the development of a multiple-choice spelling test that can be administered online and completed and scored in approximately 10 min. The positive correlations between performance on this test and measures of reading ability suggest that it captures meaningful patterns in children’s literacy development (e.g., Caravolas et al., 2001; Ehri, 1997; Kim, 2020; Kim et al., 2024; Perfetti, 2017; Swanson et al., 2003; Treiman et al., 2024). Existing spelling measures typically rely on production and therefore take 20 to 30 min to administer (e.g., New Group Spelling Test; GL Assessment, n.d.). Moreover, these measures are often embedded within extensive assessment batteries (e.g., Robertson & Wilkinson, 2017; Wechsler, 2017), which limits their practicality in resource-constrained settings. A quick and easy-to-administer test is therefore particularly valuable, as it allows large cohorts of pupils to be assessed simultaneously with minimal demands on researchers or teachers. Our spelling test is openly available, and because its items are drawn from England’s spelling curriculum (https://spellingframe.co.uk/), it is well aligned with the educational framework in the United Kingdom, making it suitable for wide use. The methodology used to develop the items and foils in our test could also be adapted to design assessments for different populations (e.g., secondary school pupils).

Finally, our paper marks the first use of the ROAR tests for single-word reading (ROAR-LDT; Yeatman et al., 2021) and sentence reading efficiency (ROAR-Sentence; Yeatman et al., 2024) with a British sample. The ROAR tests are valuable tools because they can be administered online and completed quickly; however, they were developed for and validated against U.S. pupils. In this study, we made minor changes to the tests to improve accessibility and reflect language usage in the United Kingdom, and the fact that the pattern of results across these adapted ROAR tests and our other measures mirrors that observed for U.S. pupils suggests that the adapted tests are appropriate for use with British primary school children.

To conclude, we have developed and validated two new tests of text experience, along with measures of reading and spelling skill, that are appropriate for British primary school children. The full test battery used in this study is openly available to enable quick online assessment of children’s text experience and basic literacy skills, and we hope it will serve as a valuable resource for both researchers and practitioners interested in children’s literacy development.

## Supplemental Material

sj-pdf-1-qjp-10.1177_17470218261421104 – Supplemental material for Assessing Text Experience in British Primary School Children: New Validated Title and Author Recognition Tests Supplemental material, sj-pdf-1-qjp-10.1177_17470218261421104 for Assessing Text Experience in British Primary School Children: New Validated Title and Author Recognition Tests by Holly Cooper, Maria Korochkina, Marc Brysbaert and Kathleen Rastle in Quarterly Journal of Experimental Psychology
