# Supplementary material for: Assessing Text Experience in British Primary School Children: New Validated Title and Author Recognition Tests
Source: Q J Exp Psychol (Hove). 2026 Jan 23;79(9):2337–48. doi: 10.1177/17470218261421104 (PMC13498748; doi:10.1177/17470218261421104)
Supplement: Supplementary file 1 [file sj-pdf-1-qjp-10.1177_17470218261421104.pdf]

Supplementary Material for:

Assessing text experience in British primary school children: New validated title and author recognition tests

Holly Cooper[1], Maria Korochkina[1], Marc Brysbaert[2], and Kathleen Rastle[1]

[1] Department of Psychology, Royal Holloway, University of London, United Kingdom

[2] Department of Experimental Psychology, Ghent University, Belgium

Table S1

Finalised ART and TRT item lists. The lists include real titles and authors (in black text) and foils (presented in red text). The instructions are as follows:

"You will see two lists. The first includes book titles and the second includes authors' names. Some book titles and authors' names are real and some are just made-up. Please read them carefully and tick the ones you recognise. Be careful not to tick the made-up ones. If you do not recognise a title or name, then leave the box blank."

Titles	Authors
Monkey Puzzle	Agatha Christie
When the Sky Falls	Andy Griffiths
The Highland Falcon Thief	Anne Fine
The Racehorse Who Wouldn't Gallop	Anthony Horowitz
Letters from the Lighthouse	Beatrix Potter
Of Mice and Men	Benjamin Zephaniah
The Day I Fell in to a Fairytale	C.S. Lewis
Oh The Places You'll Go	Charles Dickens
The Frog Prince	Cressida Cowell
The Boy Who Made The World Disappear	Dav Pilkey
Lord of the Flies	David Baddiel
The Explorer	David Walliams
The Girl of Ink and Stars	Dick King Smith
Refugee Boy	Dr Seuss
Giraffes Can't Dance	E. Nesbit

The Amber Spyglass	E.B. White
The Ickabog	Emma Carroll
Owl Babies	Enid Blyton
A Squash and a Squeeze	Eva Ibbotson
Rowley Jefferson's Awesome Friendly Adventure	Francesca Simon
Skulduggery Pleasant	George Orwell
Hairy Maclary from Donaldson's Dairy	Hans Christian Anderson
The Tales Of Beedle The Bard	J.K. Rowling
To Kill a Mockingbird	J.R.R. Tolkien
Journey to the River Sea	Jacqueline Wilson
Scarlet and Ivy	Jane Austen
Goodnight Mister Tom	Jeff Kinney
The London Eye Mystery	Jill Murphy
The Smartest Giant in Town	Judith Kerr
The Wizards of Once	Julia Donaldson
Funnybones	Katherine Rundell
What the Ladybird Heard	Lauren Child
Aliens Love Underpants	Lewis Carroll
Treasure Island	Liz Pichon
The Owl Who Was Afraid of the Dark	M.G. Leonard
The Extremely Embarrassing Life of Lottie Brooks	Michael Morpurgo
You're a Bad Man Mr Gum	Onjali Q. Rauf
Cat Kid Comic Club	Philip Pullman
The Giraffe and the Pelly and Me	Rick Riordan
Code Name Bananas	Roald Dahl
Murder Most Unladylike	Robert Louis Stevenson
Awful Auntie	Robin Stevens
George's Marvellous Medicine	Sophie McKenzie
Demon Dentist	Stephen King
The Boy At The Back Of The Class	Suzanne Collins
The Magic Finger	Terry Pratchett
The 13-Storey Treehouse	Tony Ross
The World's Worst Parents	A. Cowey
The Twits	P.E. Bryant
Dog Man	K. Parkes
The Midnight Kangaroo	Mansur Lalljee

A Pack of Ghosts	Paul Azzopardi
The Annoying Squirrel	Dick Passingham
The Steam Monster	M. Rushworth
The Invisible Sheep	G.B. Henning
Poor Marmot	V. Walsh
My Father's Penguin	Jane Mellanby
Niece's Dance	Kia Nobre
The Brother Library	Brian Rogers
Sweeney's Wolf	D.A. Allport
Escape Tent	Kim Plunkett
Calling on Seagulls	M. Treisman
The Mediocre Beginning	S. Millar
See You at Michael's	Nick Rawlins
The Imagination Wardrobe	D. Gaffan
The Wheel of Joy	Larry Weiskrantz
The Warrior in My Head	David Popplewell
King of the Soil	G. Claridge
The Tired Nights	Charles Spence
Tunnel of Fingers	Edmund Rolls
A Fireman Calls	Catherine Harmer
Burning Late	Mark Buckley
The Sledge Runner	Miriam Huntley
Teal Queen	Elias Maple
An Arrow to the Shed	Maeve Zawe
Dance into You	Akyiel Turner
The Gloomy Dogs	Luca Aman
The Levenson Boys	
The Catch of the Scorpion	
Until I Push	

Table S2

The table presents sentences from Yeatman et al. (2024) that we edited for our UK sample.

Original sentence	Edited sentence
Trash cans are where you store extra notebook paper.	Rubbish bins are where you store extra notebook paper.
Some houses have back yards.	Some houses have back gardens.
Soda is always brown.	Fizzy drinks are always brown.
Trash is thrown away in bathtubs.	Rubbish is thrown away in bathtubs.
Soccer is a game you play with only your hands.	Football is a game you play with only your hands.
Sweaters can be made of coal.	Jumpers can be made of coal.
Most dogs wear leashes when they go for a walk.	Most dogs wear leads when they go for a walk.
People can use cash to pay in stores.	People can use cash to pay in shops.
Moms like eating hats.	Mums like eating hats.
Stores are where you go to buy things.	Shops are where you go to buy things.
People can use credit cards to pay in stores.	People can use credit cards to pay in shops.
Trashcans can become smelly if trash is left inside too long.	Bins can become smelly if rubbish is left inside too long.
Police officers drive school busses while on duty.	Police officers drive school buses while on duty.

Table S3

The table presents the practice and experimental trial spellings used in the task. Spelling 1 is the correct spelling and spellings 2, 3, and 4 are the incorrect spelling options. The carrier sentence narrated in the task is also presented.

Trial	spelling 1 (correct)	spelling 2	spelling 3	spelling 4	Spoken sentence
practice	heart	hart	hert	haart	The **heart** pumps blood around the body.
practice	forward	forwood	forwurd	fourward	She leaned **forward** to pet the dog.
practice	library	libary	liberry	liberary	There were lots of books on the Romans in the school **library.**
practice	difficult	dificult	difficalt	dificullt	The maths test was very **difficult.**
1	immediately	imediately	immediatly	immedietly	They had to move their car **immediately** for the ambulance to pass.
2	especially	expecially	espeshly	espesially	The music was written **especially** for the film.
3	guard	gaurd	gard	guarde	Rob was working as a security **guard** near the school.
4	familiar	familear	familier	famillliar	They were **familiar** with the area and recognised the buildings.
5	soldier	soldjer	soldger	solger	Tim's great grandfather was a **soldier** in the second world war.
6	knowledge	knowlege	knolledge	nowledge	The farmer had a lot of **knowledge** about animals.
7	necessary	neccesary	neccessary	nessacery	It is **necessary** to handle scissors with care.
8	restaurant	resterant	resturant	restarent	Abbey loved the pizza at the local Italian **restaurant.**
9	pressure	presure	preshure	pressur	Sam felt great **pressure** to perform well at the concert.
10	separate	seprate	separete	separat	The clean and dirty plates were in **separate** piles.
11	naughty	norty	naughtie	nauty	The **naughty** puppy had chewed my shoe.
12	persuade	perswade	pursuade	persuaid	I tried to **persuade** my mum to let me stay up late.
13	bargain	bargian	bargin	bargan	Marie loved to find a **bargain** when she went shopping.
14	achieve	acheive	acheeve	acheave	I would like to **achieve** excellent exam results.

15	bruise	briuse	bruse	bruiz	She fell off her bike and had a **bruise** on her arm.
16	definite	definate	definat	definit	She needed a **definite** answer by Tuesday.
17	privilege	previlege	privlege	priviledge	It was a massive **privilege** to watch my team win.
18	relevant	relevint	relavant	relavent	The **relevant** information was highlighted in the textbook.
19	learn	lern	lurn	leern	Jack wants to **learn** how to play the piano.
20	certain	certun	sertain	certern	He was **certain** that he left his wallet on the desk.
